# Physical and biological dosimetric margin according to prescription method for stereotactic body radiation therapy

**DOI:** 10.1093/jrr/rrac097

**Published:** 2023-01-10

**Authors:** Daisuke Kawahara, Akito Saito, Yasushi Nagata

**Affiliations:** Department of Radiation Oncology, Institute of Biomedical and Health Sciences, Hiroshima University, Hiroshima 734-8551, Japan; Department of Radiation Oncology, Institute of Biomedical and Health Sciences, Hiroshima University, Hiroshima 734-8551, Japan; Department of Radiation Oncology, Institute of Biomedical and Health Sciences, Hiroshima University, Hiroshima 734-8551, Japan; Hiroshima High-Precision Radiotherapy Cancer Center, Higashiku‐ku Hiroshima, 732-0057, Japan

**Keywords:** stereotactic body radiation therapy (SBRT), biological dosimetric margin (BDM), biological conversion factor (BCF), marginal prescription method, point prescription method

## Abstract

This study aimed to expand the biological conversion factor (BCF) model, which converts the physical dosimetric margin (PDM) to the biological dosimetric margin (BDM) for point prescription with 3-dimensional conformal radiation therapy (3DCRT) and the marginal prescription method with volumetric-modulated arc radiotherapy (VMAT). The VMAT of the marginal prescription and the 3DCRT of the point prescription with lung stereotactic body radiation therapy (SBRT) by using RayStation were planned. The biological equivalent dose (BED) for a dose per fraction (DPF) of 3–20 Gy was calculated from these plans. The dose was perturbed with the calculation using a 1-mm step isocenter shift. The dose covering 95% of the target was greater than or equal to 90% of the prescribed physical dose, and the BED were defined as the PDM and BDM, respectively. The BCF was created as a function of the DPF. The PDM and BDM for all DPFs were larger with the point prescription method than with the marginal prescription method. The marginal prescription method with a 60% isodose line had a larger PDM and BDM. The BCF with the point prescription was smaller than that with the marginal prescription in the left–right (LR), anterior–posterior (AP) and cranio–caudal (CC) directions. In the marginal prescription method, the 60% isodose line had a higher BCF. In conclusion, the improved BCF method could be converted to BDM for point prescription with 3DCRT and marginal prescription method with VMAT, which is required for stereotactic radiation therapy in radiobiology-based treatment planning.

## INTRODUCTION

Dose-volume histograms (DVH) and physical dose distributions are known evaluation methods in treatment planning. Previous radiobiological and clinical studies have reported that the same total physical dose through different treatment schedules produces different biological results [1, 2]. This biological effect is considered in biological effective dose (BED) modeling, which is an effective tool for different fraction schemes [3]. The calculation of the BED has been commonly used with the linear-quadratic (LQ) model. The BED depends on the treatment fraction and dose per fraction (DPF). The calculation of the BED is required in stereotactic body radiation therapy (SBRT), which uses hypo-fractionation [1].

Previously, we proposed a dosimetric margin (DM) that involved the effects of dose perturbation caused by setup errors [4]. The DMs of the physical and biological dose distributions using the LQ model [5] were defined as the physical dosimetric margin (PDM) and biological dosimetric margin (BDM), respectively. Moreover, the biological conversion factor (BCF) was proposed to provide an appropriate DM for BED-based treatment planning for each fractionation scheme. However, the proposed BCF model used a marginal prescription at the 80% isodose level with 3-dimensional conformal radiotherapy techniques (3DCRT).

3DCRT was proposed to deliver a uniform dose to a volume; thus, the dose is often prescribed at a specific point [6]. Moreover, volumetric-modulated arc radiotherapy (VMAT), a novel radiotherapy technique, improves radiotherapy plan dose distribution over 3DCRT [2]. Chan *et al.* reported that the target coverage with VMAT was superior to that with 3DCRT [2]. The dose prescription for SBRT was described in the International Commission on Radiation Units and Measurements (ICRU) report 91 [7]. The ICRU report 91 pointed out the importance of planning target volume (PTV) dose escalation. In particular, the dose prescription method has been moved to the marginal prescription for SBRT [3, 8].

In the current study, we evaluated the PDM and BDM for VMAT lung SBRT using point prescription and marginal prescription methods. Moreover, we proposed an improved BDM model for point prescriptions with 3DCRT and a marginal prescription method with VMAT.

## MATERIALS AND METHODS

**Fig. 1:**
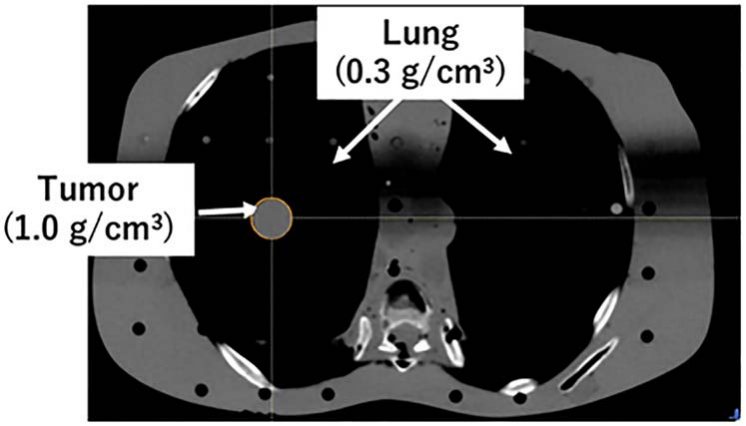
Locations of the target evaluated in the current study. The virtual sphere target was set to the center positions of the right lung. The radius of the tumor was 1.0 cm.

An anthropomorphic phantom, RANDO (Phantom Laboratory, Salem, NY), was used. Computed tomography (CT) scan was conducted using a 16-detector row spiral CT scanner (LightSpeed RT16, GE Healthcare, UK). A CT scan was conducted using a 16-detector row spiral CT scanner (LightSpeed RT16, GE Healthcare, UK). The scan was reconstructed with a slice thickness and slice interval of 2.0 mm. The treatment planning system used was a RayStation ver. 6.0 (RaySearch Medical Laboratories AB, Sweden). In treatment planning, the virtual tumor was inserted into the anthropomorphic phantom, as shown in Fig. 1. The virtual tumor was defined as a water-equivalent sphere with a radius of 1.0 cm. The electron densities of the tumor and lung were assigned to 1.0 and 0.3 g/cm^3^, respectively. The virtual tumor was defined as the gross tumor volume (GTV), and the clinical target volume (CTV) margin was 0 mm around the GTV in the left–right (LR), anterior–posterior (AP) and cranio–caudal (CC) directions. The current study assumed that breath control was accomplished through breath-hold. A PTV margin was added at 5 mm from the CTV in the LR, AP and CC directions. We used 6-MV flattening filter-free beams with the TrueBeam linear accelerator (Varian Medical Systems, USA). The 3DCRT plan was created using four coplanar and four noncoplanar beams. The collimator angle was fixed at 0°. The isocenter was prescribed with 48 Gy in four fractions (‘IC prescription’). The VMAT plan was created using coplanar beams. The gantry angle was set at 0°–180°, counterclockwise. The collimator and couch angles are fixed at 10° and 0°, respectively. A dose of 48 Gy in four fractions was prescribed to the *D*_95%_ of the PTV. The DPF was varied in the range of 3–20 Gy for the BED calculation [9–15]. The details of DPF and fraction number were 3 Gy × 15 fractions [9], 5 Gy × 10 fractions [9], 6 Gy × 10 fractions [10], 7 Gy × 5 fractions [10], 7.5 Gy × 8 fractions [10], 8 Gy × 3 fractions [11], 10 Gy × 5 fractions [12], 12 Gy × 4 fractions [13, 14], 15 Gy × 3 fractions [15], 18 Gy × 3 fractions [11] and 20 Gy × 3 fractions [11]. For the marginal prescription method, the PTV was covered by a 60–80% prescription isodose volume. The collapsed cone convolution superposition algorithm calculates the dose as dose-to-medium.

**Fig. 2:**
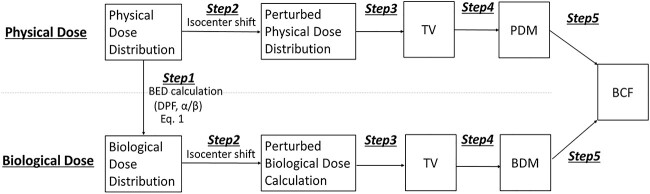
Process of calculating the BDM and PDM, which was referred from previous our study [4].

The process for calculating the PDM and BDM was proposed in our previous study, as shown in Fig. 2 [4]. The treatment planning system used was RayStation (RaySearch, Sweden). In step 1, the BED distribution was calculated from the physical dose distribution using the LQ model. The BED was recalculated using:


(1) \begin{equation*} \mathrm{BED}= nd\left(1+\frac{d}{\alpha /\beta}\right), \end{equation*}

where $\alpha /\beta$ is the repair capacity of the cells, *n* is the number of treatment fractions, and *d* is the DPF. In the current study, $\alpha /\beta$ was set to 3 Gy for normal tissue and 10 Gy for the tumor, as is accepted for conventional fractionation [16].

### Correlation of the biological and PDM for point and marginal prescriptions

**Fig. 3:**
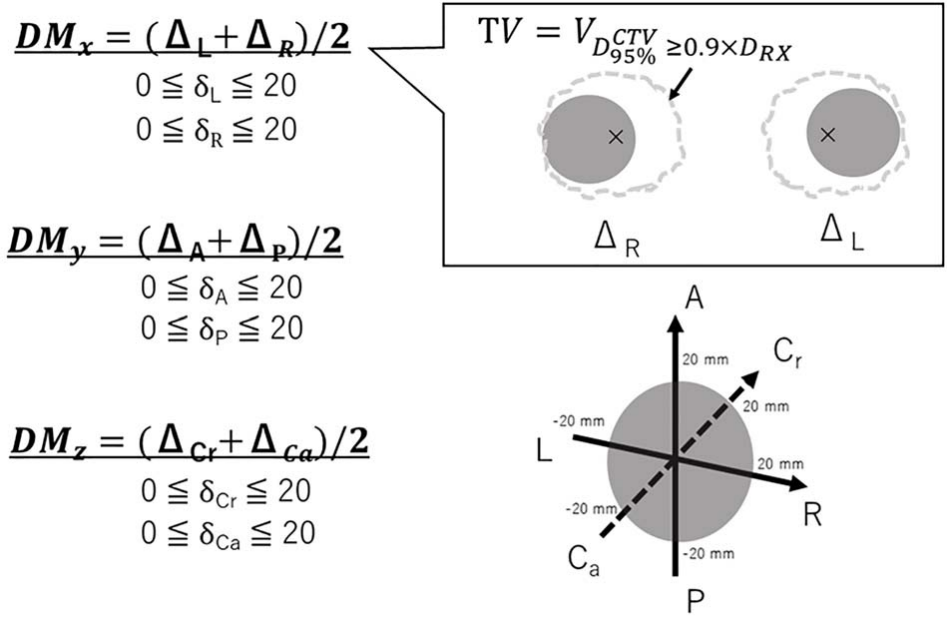
Illustration of the TV and DM, which was referred from previous our study [4].

Figure 3 illustrates the DM and treated volume (TV) proposed in a previous study [4]. Then, the isocenter was shifted from −20 to 20 mm in 1-mm steps along the LR, AP and CC directions in RayStation (step 2). In step 3 and step 4, the TV was derived, and the PDM and BDM were calculated from TV and CTV. Finally, the conversion from the PDM to BDM is defined as the BCF model (step 4).

### Biological conversion factor

The calculation process for the DM was based on a previous study (equations 3–8) [4]. The BCF was defined as the ratio between the BDM and the PDM:


(2) \begin{equation*} \mathrm{BCF}=\frac{\mathrm{BDM}}{\mathrm{PDM}}. \end{equation*}

The BCF is modeled by fitting using the following two functions of $d/(\alpha /\beta )$.


(3) \begin{equation*} {\mathrm{BCF}}_{point}=A\ \ln \left(\frac{d}{\alpha /\beta}\right)+B, \end{equation*}

(4) \begin{equation*} {\mathrm{BCF}}_{marginal}=\left(C\times ID+D\right)\ln \left(\frac{d}{\alpha /\beta}\right)+\left(\mathrm{E}\times ID+F\right), \end{equation*}

Equation 4 improved equation 3, which was used for the point prescription, was added to the function of the isodose level (*ID*) in the range of 60-80%. *A*, *B, C* and *D* are the fitting parameters determined using the least-squares method. The fitting equation was modified from our previous model to fit the points and marginal prescriptions. Moreover, tumor density may affect the dose distribution, which leads to the robustness of the BCF. BCFs with electron densities of 0.4, 0.7 and 1.0 g/cm^3^ were compared.

## RESULTS

### Correlation of the biological and PDM for point and marginal prescriptions

**Fig. 4:**
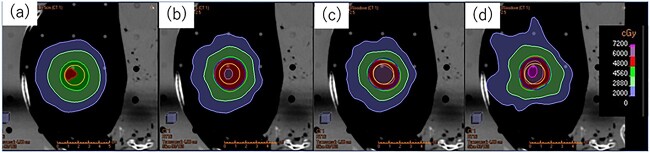
Physical dose distribution for IC prescription in 3DCRT (a) (left) and marginal prescription at 60% (b), 70% (c) and 80% (d) isodose line in VMAT (right).

**Fig. 5:**
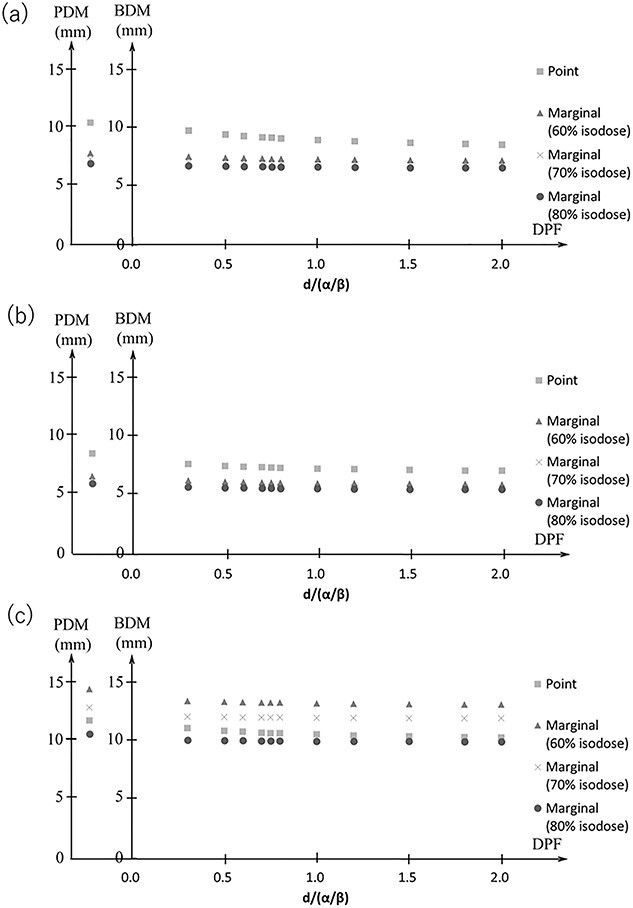
PDM and BDM of *D*_95%_ in LR (a), AP (b) and CC (c) directions for IC and marginal prescriptions.

Figure 4 shows the dose distribution for the point and marginal prescription at the 60–80% isodose line. Figure 5 shows the BDM and PDM for the *D*_95%_ of the CTV for IC and marginal prescriptions, respectively. The DPF ranged from 3 to 20 Gy, with $\alpha /\beta$ = 10 Gy. The BDM and PDM for the point prescription were larger than those for the marginal prescription at the 60–80% isodose line in all directions. The maximum difference in the PDM between the point prescription and the marginal prescription was 5.0 mm in the LR direction, 2.8 mm in the AP direction and 3.8 mm in the CC direction. The maximum difference of the BDM between the point prescription and the marginal prescription for the DPF of 3–20 Gy was 4.4 mm in the LR direction, 2.4 mm in the AP direction and 4.0 mm in the CC direction.

For the marginal prescription, the BDM and PDM at the smaller isodose line had larger PDM and BDM. The maximum difference of the PDM was 2.4 mm in the LR direction, 0.8 mm in the AP direction and 6.5 mm in the CC direction. The maximum difference of the BDM for the DPF of 3–20 Gy was 2.2 mm in the LR direction, 1.0 mm in the AP direction and 6.3 mm in the CC direction.

### Biological conversion factor

**Fig. 6:**
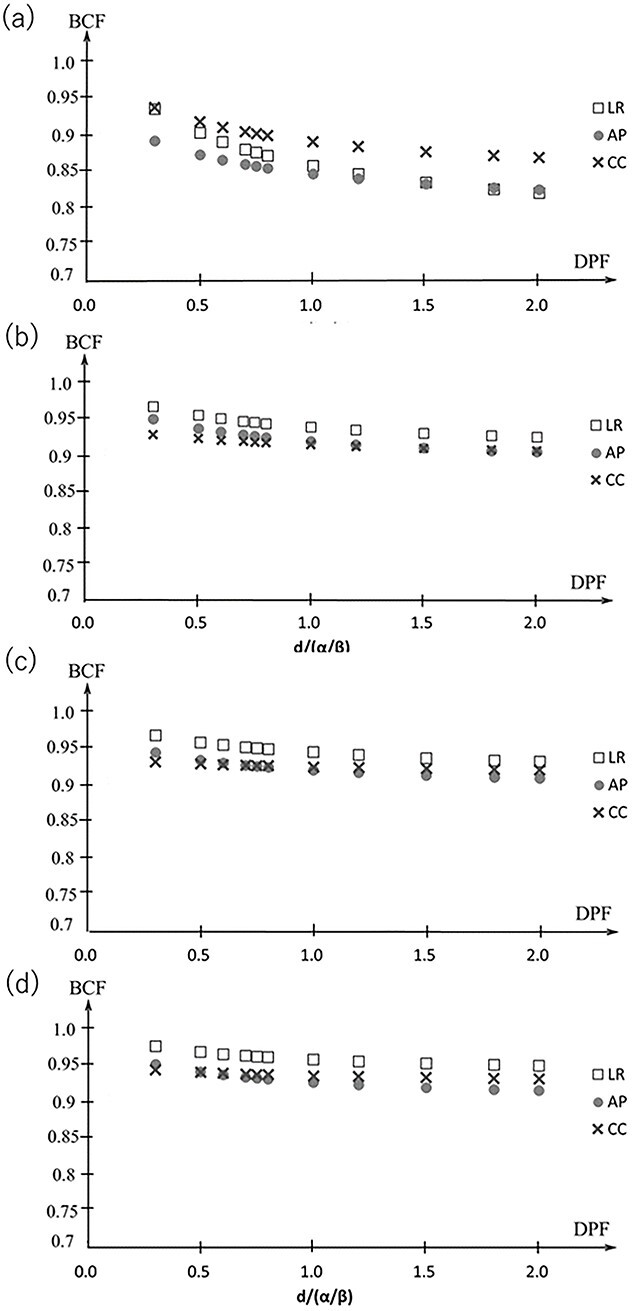
BCF for the DPF of 3–20 Gy with $\alpha /\beta$ = 10 Gy in the LR, AP and CC directions for the point prescription (a) and the marginal prescription at 60% isodose line (b), 70% isodose line (c) and 80% isodose line levels (d).

**Fig. 7:**
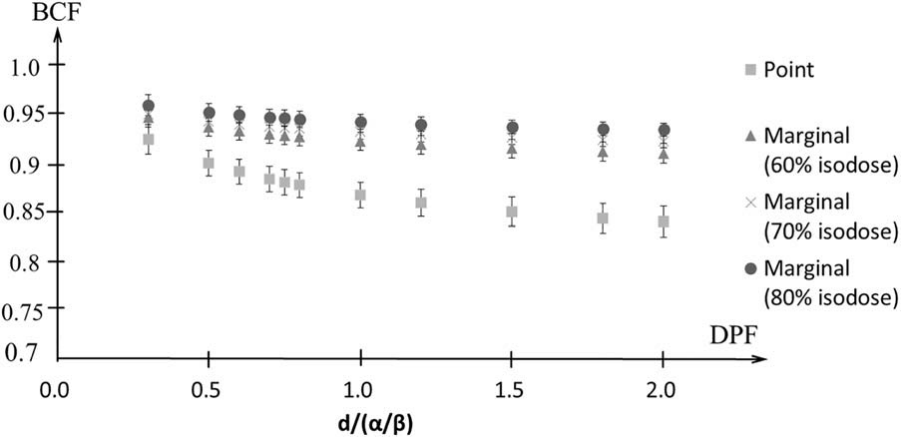
Average and standard error (SE) of the BCF for the DPF of 3–20 Gy for the point and marginal prescriptions.

**Fig. 8:**
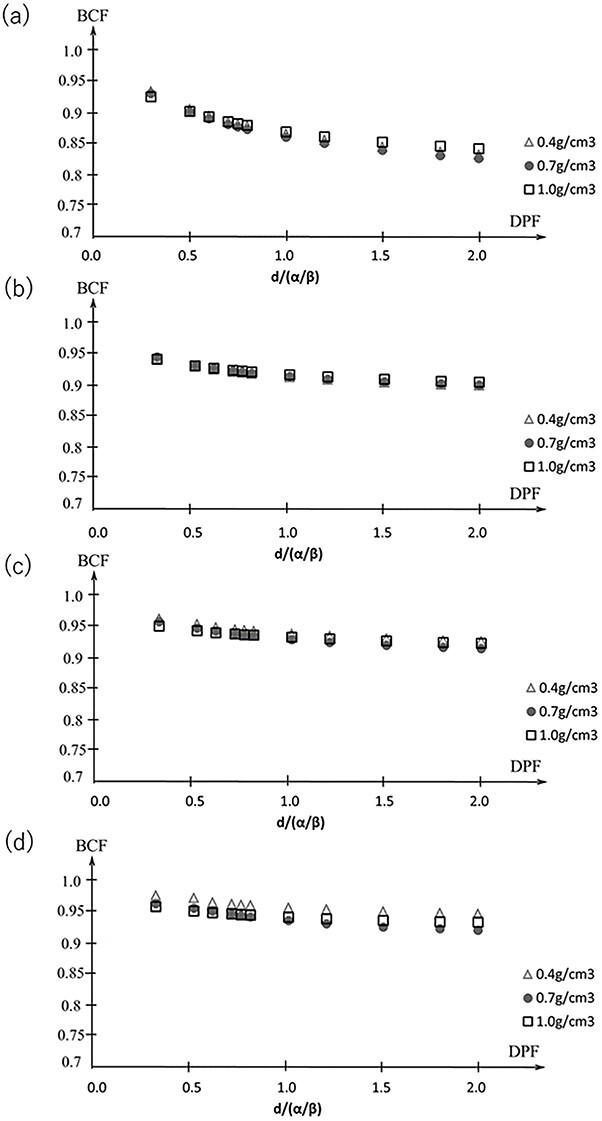
Average BCF for the DPF of 3–20 Gy with tumor densities of 0.4, 0.7 and 1.0 g/cm^3^ for the point and marginal prescriptions.

**Fig. 9:**
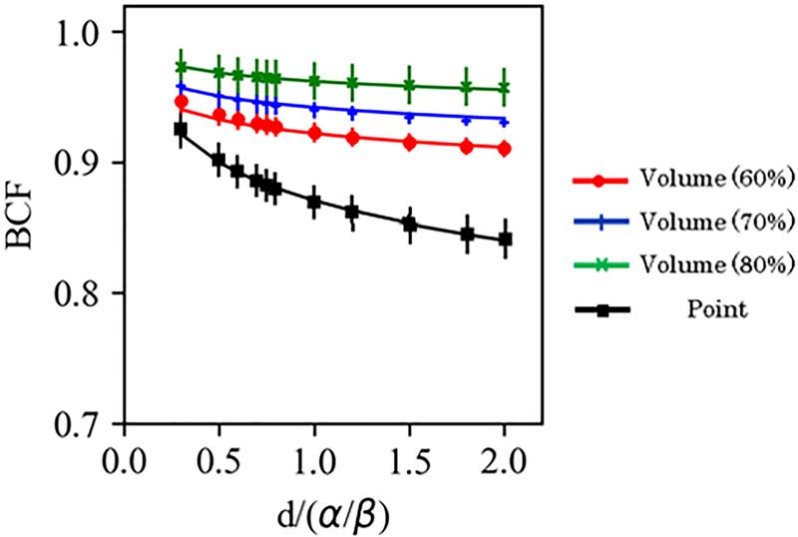
BCF with the $d/(\alpha /\beta)$. The closed squares represent the data of the BCF for the point prescriptions. The closed circles, plus symbols and cross symbols represent the data of the BCF at 60%, 70% and 80% isodose line in the marginal prescriptions, respectively. Error bars represent the SEM. The line curves are the results of the fitting using equations (3) and (4) for the point and marginal prescriptions.

Figure 6 shows the BCF for a DPF of 3–20 Gy with $\alpha /\beta$ = 10 Gy. The maximum difference in the BCF for the point prescription in the LR, AP and CC directions was within 0.05. For the marginal prescription, the maximum difference of the BCF of the DPF at 3–20 Gy was within 0.04 at 60% isodose line, 0.03 at 70% isodose line and 0.03 at 80% isodose line. Figure 7 shows the average and standard error of the mean (SEM) of the BCF in the LR, AP and CC directions. The SEM was within 0.02 for both the point prescription and the marginal prescription at 60–80% isodose lines. The differences in BCF due to $\alpha /\beta$ were not significant in the LR, AP and CC directions. Thus, the data in all the directions were combined for fitting. The BCF according to electron density is shown in Fig. 8. The difference of the BCF with the electron densities of 0.4, 0.7, and 1.0 g/cm^3^ was within the SEM of the BCF in LR, AP and CC directions. Figure 9 shows the measurement data and the fitted curve for the point prescription and marginal prescription of 60–80% isodose lines. The parameters of the BCF are shown in Table 1 for point prescription and Table 2 for marginal prescription. The gradient from lower to higher DPF was larger for the point prescription than for the marginal prescription.

## DISCUSSION

Generally, the PTV margin is defined as the region in which the CTV covers the prescribed dose with a specified probability of geometrical variations [17]. The proposed DM involves dose perturbation. Previously, we proposed a DM that involved the effects of dose perturbation caused by setup errors in the clinic [4]. In a previous study, a novel scheme involving the biological effects of PDM was introduced as a point prescription method with 3DCRT. We showed that the BDM was significantly larger than the PDM, and proposed the BCF, which converts the PDM to BDM for lung cancer patients in clinical settings. The current study evaluated the PDM and BDM for the point prescription method, which is frequently used for 3DCRT, and the marginal prescription method with VMAT, which is a novel radiotherapy technique. The PDM was slightly larger than the BDM for all isodose lines of 60–80% in marginal prescriptions. The gradient of the BCF due to the DPF was significantly larger for point prescription. In general, the evaluation of the treatment plan is performed using a physical dose. The BDM was smaller for a larger DPF, which indicates a larger margin is required for a higher DPF.

The BCF model was improved to be adequate for point prescription with 3DCRT and marginal prescriptions of 60–80% isodose lines with VMAT. The previous BCF model was developed separately for the transverse and longitudinal directions. However, there was no significant difference in the direction of the setup error in the BCF at the 60–80% isodose line in the marginal prescription. Thus, the BCF model was created without any difference in the direction of setup error.

Kartutik *et al.* compared 3DCRT and intensity-modulated radiation therapy (IMRT) in lung SBRT [18]. They concluded that conformity and homogeneity with IMRT were superior to those with 3DCRT. However, dosimetric target coverage, such as setup errors, was not considered. The current study showed that the biological dose difference and the BCF for the setup error were larger when the PDM in 3DCRT was equal to that in VMAT. The BCF was larger with a higher isodose line for the marginal prescription method because the dose gradient was lower. From the above, the target robustness in the physical and biological doses was higher for a higher isodose line in marginal prescription with VMAT.

**Table 1:** The fitting parameters using equation (3) for the point prescription

Parameter	Value	SD
*A*	−6.84e-02	7.01e-02
*B*	0.80	6.03e-02

**Table 2:** The fitting parameters using equation (4) for the marginal prescriptions

Parameter	Value	SD
*C*	4.51e-04	4.20e-05
*D*	−4.66e-02	3.06e-05
*E*	1.86e-03	3.67 e-05
*F*	8.11e-01	2.49e-05

The current study has three limitations. The first limitation is that other parameters, such as tumor location and tumor volume, must be considered. However, our previous study investigated the correlation between tumor volume and PDM in 10 patients who underwent lung SBRT. The results showed a weaker correlation, indicating that the target volume does not affect the PDM and BDM [4]. Moreover, our previous study analyzed patients with various tumor locations [4]. In the current study, the standard error did not differ significantly between the patient and the phantom. Thus, the tumor location may not strongly affect PDM and BDM. However, future studies will be performed to investigate the PDM and BDM change with and without scatter material, such as the chest wall around the tumor, to enhance the robustness of our BDM model. Second, the BDM is calculated on the normal tissue, such as the BED for the normal lung volume and the acceptable margin in clinical practice. Third, the influence of random error should also be considered. The current study assumed a breath-hold case and considered only systematic errors. Respiratory motion is a significant issue for radiation therapy and is affected by numerous patient-related issues and chemotherapeutic agents, including coexisting pulmonary disease, concomitant infection and cachexia [19, 20]. There are various approaches to the motion management of lung tumors, including imaging, respiratory tracking, breath-hold techniques and abdominal compression. Thus, if random respiratory tumor motion is considered, the PDM and BDM could change. The various prescribed doses for patient treatment should be determined not only by the setup margin and organ motion but also by the difference in the biological effect. The BDM is advantageous for evaluating the relative BED coverage. The expanded BCF model can be used in clinical treatment and prescription methods. Further studies will be conducted to evaluate the PDM and the BDM with random errors and respiratory motion.

## CONCLUSION

An improved scheme for directly estimating the BDM using the BCF corresponding to the prescription method was proposed. The improved BCF method can convert the BDM for point prescription with 3DCRT and the marginal prescription method with VMAT.

## CONFLICT OF INTEREST

The authors declare they have no conflicts of interest.

## References

[1] Blomgren H, Lax I, Naslund I et al. Stereotactic high dose fraction radiation therapy of extracranial tumors using an accelerator. Clinical experience of the first thirty-one patients. Acta Oncol 1995;34:861–70. 7576756 10.3109/02841869509127197

[2] Chan O, Lee M, Hung A et al. The superiority of hybrid-volumetric arc therapy (VMAT) technique over double arcs VMAT and 3D-conformal technique in the treatment of locally advanced non-small cell lung cancer – a planning study. Radiother Oncol 2011;101:298–302. 21907438 10.1016/j.radonc.2011.08.015

[3] Fowler JF . The linear-quadratic formula and progress in fractionated radiotherapy. Br J Radiol 1989;62:679–94. 2670032 10.1259/0007-1285-62-740-679

[4] Kawahara D, Saito A, Ozawa S et al. Assessment of biological dosimetric margin for stereotactic body radiation therapy. J Appl Clin Med Phys 2020;21:31–41. 32141684 10.1002/acm2.12843 PMC7170295

[5] Beyzadeoglu M, Ozyigit G, Ebruli C. Basic Radiation Oncology. Berlin Heidelberg: Springer-Verlag, 2010, 145–73.

[6] Douglas J. ICRU . 50 Prescribing, recording, and reporting photon beam therapy. In: International Commission on Radiation Units and Measurements ICRU Report 50. Bethesda, MD, 1993.

[7] Wilke L, Andratschke N, Blanck O et al. ICRU report 91 on prescribing, recording, and reporting of stereotactic treatments with small photon beams: statement from the DEGRO/DGMP working group stereotactic radiotherapy and radiosurgery. Strahlenther Onkol 2019;195:193–8. 30649567 10.1007/s00066-018-1416-x

[8] Timmerman RD, Michalski J, Fowler J et al. A Phase II Trial of Stereotactic Body Radiation Therapy (SBRT) in the Treatment of Patients with Medically Inoperable Stage I/II Non-small Cell Lung Cancer, Protocol 0236. Philadelphia: RTOG, 2006.

[9] Shien G, Jie Y, Jiang W et al. Stereotactic body radiation therapy for centrally-located lung tumors. Oncol Lett 2014;7:1292–6. 24944711 10.3892/ol.2014.1815 PMC3961379

[10] Onimaru R, Shirato H, Shimizu S et al. Tolerance of organs at risk in small-volume, hypofractionated, image-guided radiotherapy for primary and metastatic lung cancers. Int J Radiat Oncol Biol Phys 2003;56:126–35. 12694831 10.1016/s0360-3016(03)00095-6

[11] Timmerman R, Papiez L, McGarry R et al. Extra cranial stereotactic radioablation: results of a phase I study in medically inoperable stage I non-small cell lung cancer. Chest 2003;124:1946–55. 14605072 10.1378/chest.124.5.1946

[12] Uematsu M, Shioda A, Suda A et al. Computed tomography-guided frameless stereotactic radiotherapy for stage I non-small cell lung cancer: a 5-year experience. Int J Radiat Oncol Biol Phys 2001;51:666–70. 11597807 10.1016/s0360-3016(01)01703-5

[13] Nagata Y, Takayama K, Matsuo Y et al. Clinical outcomes of a phase I/II study of 48 Gy of stereotactic body radiotherapy in 4 fractions for primary lung cancer using a stereotactic body frame. Int J Radiat Oncol Biol Phys 2005;63:1427–31. 16169670 10.1016/j.ijrobp.2005.05.034

[14] Taremi M, Hope A, Dahele M et al. Stereotactic body radiotherapy for medically inoperable lung cancer: prospective, single-center study of 108 consecutive patients. Int J Radiat Oncol Biol Phys 2012;82:967–73. 21377293 10.1016/j.ijrobp.2010.12.039

[15] Wulf J, Haedinger U, Oppitz U et al. Stereotactic radiotherapy for primary lung cancer and pulmonary metastases: a noninvasive treatment approach in medically inoperable patients. Int J Radiat Oncol Biol Phys 2004;60:186–96. 15337555 10.1016/j.ijrobp.2004.02.060

[16] Bentzen SM, Dörr W, Gahbauer R et al. Bioeffect modeling and equieffective dose concepts in radiation oncology – terminology, quantities and units. Radiother Oncol 2012;105:266–8. 23157980 10.1016/j.radonc.2012.10.006

[17] van Herk M, Remeijer P, Rasch C, Lebesque JV. The probability of correct target dosage: Dosepopulation histograms for deriving treatment margins in radiotherapy. Int J Radiat Oncol Biol Phys 2000;47:1121–35. 10863086 10.1016/s0360-3016(00)00518-6

[18] Kartutik K, Wibowo WE, Pawiro SA et al. Comparison of radiotherapy dosimetry for 3D-CRT, IMRT, and SBRT based on electron density calibration. J Phys 2016;694(1):012017.

[19] Shirato H, Seppenwoolde Y, Kitamura K et al. Intrafractional tumor motion: lung and liver. Semin Radiat Oncol 2004;14:10–8. 14752729 10.1053/j.semradonc.2003.10.008

[20] Waghorn BJ, Shah AP, Rineer JM et al. A margin-based analysis of the dosimetric impact of motion on step-and-shoot IMRT lung plans. Radiat Oncol 2014;9:46. https://www.ncbi.nlm.nih.gov/pmc/articles/PMC3922402/24499602 10.1186/1748-717X-9-46 PMC3922402

